# Urinary biomarkers improve prediction of AKI in pediatric cardiac surgery

**DOI:** 10.3389/fped.2025.1515210

**Published:** 2025-03-13

**Authors:** Oscar T. Deal, Thomas Mitchell, Amy G. Harris, Kelly Saunders, Julie Madden, Carrie Cherrington, Karen Sheehan, Mai Baquedano, Rusununguko Kanyongo, Giulia Parolari, Kirsty Phillips, Serban Stoica, Massimo Caputo, Francesca Bartoli-Leonard

**Affiliations:** ^1^ Bristol Medical School, Faculty of Health Sciences, University of Bristol, Bristol, United Kingdom; ^2^ Bristol Heart Institute, University Hospital Bristol and Weston NHS Foundation Trust, Bristol, United Kingdom; ^3^ Cardiovascular Research Unit, University of Cape Town, Cape Town, South Africa; ^4^ Christiaan Barnard Division for Cardiothoracic Surgery, University of Cape Town, Cape Town, South Africa

**Keywords:** congential heart disease, acute kidney injury, biomarker, cardiac surgery, neonatal, paediatric surgery

## Abstract

Acute kidney injury (AKI) is a common postoperative complication of paediatric congenital heart disease (CHD) surgery, associated with increased morbidity and mortality. Current diagnostic approaches are unreliable in the early postoperative period, delaying diagnosis and treatment. This study investigates the efficacy of inflammatory and renal biomarkers in the early detection of postoperative AKI in paediatric CHD surgery patients. Biomarkers were assessed in urine and serum samples collected pre- and 24 h postoperatively from paediatric patients (median age 27 weeks) undergoing corrective CHD surgery (*n* = 76). Univariate and subsequent multivariate regression analysis with least absolute shrinkage and selected operator (LASSO) regularisation was performed to identify key predictors stratified by AKI diagnosis at 48 h. Significant biomarkers were included in a compound regression model which was evaluated through receiver operator curve analysis. Internal validation of the models was carried out through bootstrapping. Postoperative urine concentrations of interleukin-18 were significantly higher in those with postoperative AKI (*p* = 0.015), whereas uromodulin concentrations were lower (*p* = 0.010). Uromodulin, interleukin-18, and serum Fatty Acid Binding Protein 3 were associated with AKI (*p* = 0.011, 0.040, 0.042 respectively), with uromodulin and interleukin-18 performing strongly in a compound model withstanding LASSO regularisation, demonstrating an area under the curve of 0.899, sensitivity of 0.741, and specificity of 0.913. Urine uromodulin and interleukin-18 can be used to accurately predict postoperative AKI when measured at 24 h after surgery. Prompt recognition of postoperative AKI would facilitate early intervention, potentially mitigating the most severe consequences of renal injury.

## Introduction

Postoperative acute kidney injury (AKI) is a common complication of pediatric congenital heart surgery, with an incidence of approximately 35% of cases (1). Prompt recognition of individuals at risk of pediatric cardiac surgery associated-AKI (pCSA-AKI) is essential, as pCSA-AKI is independently associated with prolonged intensive care unit (ICU), mechanical ventilation time, and in-hospital mortality (2–4). Furthermore, even the lowest grade of pCSA-AKI is associated with increased peri-operative costs in hospital (5, 6), highlighting the need for a robust and rapid diagnostic protocol. Despite the prevalence and clinical significance of pCSA-AKI, several different diagnostic criteria exist; including pRIFLE, and KDIGO (5–7). These approaches rely on the collection of sequential serum creatinine (SCr) to detect a clinically significant rise, or the reduction in urine output to diagnose pCSA-AKI. The use of SCr as a marker of renal injury has limitations, particularly in the detection of mild damage, which can be critical in the youngest patients.

Through emerging basic and translational findings, dysregulation of the immune system has been implicated in the development of AKI pathogenesis, with changes in numerous biomarkers detected during AKI which may be able to delineate the poorly understood AKI mechanisms. Critically, the pleiotropic cytokine IL-6 has been observed to upregulate during the development of AKI in response to hypoxia and tissue injury (8, 9). Moreover, pro-inflammatory IL-18 has been proposed to mediate ischemic tubular injury, exhibiting regulatory control in a variety of autoimmune and metabolic syndromes (10). Transporter proteins including fatty acid binding protein 1 and 3 (FABP1, FABP3), considered markers of renal tubular injury and reduced glomerular filtration rate in response to ischemia or oxidative stresses have been associated with AKI in meta-analysis studies, however limited experimental knowledge has been validated (11, 12). Finally, uromodulin; the most abundant protein in human urine, which regulates water and salt metabolism has been suggested as a prognostic indicator for AKI in adults (13). However until now, the predictive ability of these biomarkers has not been assessed in a cohort of the youngest pediatric cardiac surgery patients, who are the most clinically vulnerable, with the highest rates of 30-day postoperative mortality (14) and thus represent a significant gap in our clinical understanding.

## Methods

### Study population

Plasma and urine samples were collected from consecutive series patients (*n* = 76) 24 h pre- and 24 h post- corrective cardiac surgery at Bristol Royal Hospital for Children following informed consent within the Outcome Monitoring after Cardiac Surgery of Paediatrics (OMACp) study (NHS REC 19/SW/0113) in accordance with the declaration of Helsinki. All samples were taken from patients between the age of 0 and 4 years old with all patients undergoing cardio-pulmonary bypass (CPB). Standard cardiopulmonary bypass as reported elsewhere was used [ISRCTN13467772]. Surgeons favoured mild, as opposed to moderate/deep, hypothermia and use selective cerebral perfusion in arch repair cases. Patients with suspected or diagnosed genetic pathologies were excluded. No patients received ECMO post surgery, with 8 receiving peritoneal dialysis. No mortality was recorded. Blood samples and clinical parameters used in downstream analysis were taken at the same time. Clinical data, pre- and postoperative echocardiography and clinical diagnosis were reviewed for all patients. Exclusion criteria were a recent or previous kidney transplant or dialysis, history of kidney disease, organ transplantation or suspected or diagnosed genetic syndromes; DiGeorge, Downs, Turners, Noonan and Williams.

### Sample collection & analysis

Samples were collected pre- and 24 h post- operatively. All clinical measures used in downstream analysis were collected at the same timepoint. AKI was defined as acute kidney injury of any stage as predefined by the KDIGO criteria at 48 h postoperatively (7). Within urine, IL-6, IL-18, Neutrophil gelatinase-associated lipocalin (NGAL) and Matrix Metallopeptidase 7 (MMP7) were assessed using the LuminexÒ assay (LXSAHM-04) according to manufacturer's instructions. FABP1, FABP3 and uromodulin were assessed via the DuoSetÒ ELISA (DY9465, DY1678 and DY5144 respectively) according to manufacturer's instructions. Within plasma, FABP3 and N-terminal pro B-type natriuretic peptide (NTproBNP) were assessed via the DuoSet ELISA (DY1678 and DY3604 respectively) and Creatine Kinase Myocardial-Band (CKMB) was assessed via Human ProcartaPlex simplex kit (EPX010-12264-901) according to manufacturer's instructions.

### Statistical analysis

All statistical analysis was performed in R statistical software (the R foundation; version 4.3.2), utilising the supplementary packages “dplyr” (v1.1.2) (15) and “ggplot2” (v3.4.4) (16). Data is displayed as mean ± standard deviation or median with interquartile range for normally distributed variables and non-normally distributed continuous variables respectively. The two-sided *z*-test and Mann–Whitney *U*-test were used to compare distribution of continuous variables between age-matched cohorts with and without AKI as appropriate. Comparison of tertile medians was performed using the Kruskal–Wallis test. Survival analysis including Cox proportional hazards modelling was performed using the “survival” package (v3.6–4) (17).

### Imputation of missing data

Missing confounding variable data (height, weight, BSA, pre-operative SCr, cross-clamp time) was imputed using the predictive mean matching approach within the “mice” package (v3.16.0) (18) for inclusion in the regression models, using a multiple imputation number of 5. The percentage imputed data for each variable was as follows; height 5.2%, weight 1.3%, BSA 5.2%, cross-clamp time 9.2%, pre-operative creatinine 32.8%.

### Regression analysis

Univariate logistic regression analysis was performed to assess the association between each clinical biomarker and presence of pCSA-AKI. Single biomarker models were then adjusted for age (in weeks) and sex initially (co-variate group 1), then using a composite of confounding variables [age (in weeks), sex, body surface area (BSA; calculated using the Mostellar method), cross-clamp time, CPB time, preoperative SCr level, and 24 h postoperative change in SCr; co-variate group 2]. Collinearity was assessed through the variance inflation factor (VIF), within the “olsrr” package. Collinearity between age and BSA was VIF of 2.243 with a tolerance of 0.446 and between cross clamp and bypass time VIF was 2.389 and a tolerance of 0.419. Overall collinearity of the model was acceptable with no significant collinearity present. Clinical biomarkers with a significance of *p* < 0.1 in the composite-adjusted models (urine IL-18, urine uromodulin, serum FABP3) were selected for inclusion in least absolute shrinkage and selected operator (LASSO) regression analysis. LASSO regression employs a procedure by which coefficients of redundant variables are constrained to zero by applying a penalty; defined here as 3-fold cross validation and selecting the lambda with the smallest cross validation error. Stability of the model was calculated via 1,000 bootstrapped resampling, before the biomarker was included in the multivariate model. Regression analyses were performed using the “glmnet” package (v4.1.8) (19).

Performance of the LASSO regression models was assessed through receiver operating characteristic area under the curve (ROC-AUC) and Brier scores utilising the packages “pROC” (v1.18.5) (20) and “DescTools” (v0.99.53) (21). AUC, threshold, sensitivity, and specificity were calculated for the univariate and multivariate models. Bootstrapping of the univariate and multivariate LASSO models was performed at a 95% confidence interval with a bootstrap replicate number of 1,000. Tertiling of IL-18 and uromodulin was performed by dividing the range of biomarker concentrations by 3 and stratifying each individual based on their concentration tertile. Poisson regression analysis was performed to assess the relationship between ICU stay length and biomarker tertiles. Model 1 was adjusted for co-variate group 1 and Model 2 was adjusted for co-variate group 2.

### Decision curve analysis

The clinical utility of the compound model was assessed using decision curve analysis (DCA) through comparison with a 48-hour postoperative delta SCr model representing current methods of pCSA-AKI diagnosis. Briefly, a DCA provides insights into model performance for decision making, taking into account the known information. If the threshold is set at 25% (the conservative estimated incidence of post operative AKI) then the increase in positive diagnosis of AKI in this population can be read off the *y*-axis. DCA was performed using the “dcurves” package (v0.4.0) (22).

## Results

Biomarkers known to be associated with renal injury and inflammation were screened within the patient cohort at 24 h preoperatively (baseline) and 24 h postoperatively. Of the 76 children undergoing cardiac surgery (Table 1) 43 patients (56.6%) met the outcome measure of pCSA-AKI (defined as AKI of any stage within 48 h postoperatively as described by the KDIGO criteria) with 19% of those receiving peritoneal dialysis.

**Table 1:** Clinical characteristics of paediatric patients undergoing reparative congenital heart surgery stratified by occurrence of acute kidney injury.

Variable/characteristic	no AKI	AKI	*p*-value
	(*n* = 33)	(*n* = 43)	
Age (weeks)	31 (18.00–45.00)	17 (2.00–36.50)	0.027*
Female (*n*, %)	17 (59%)	9 (26%)	0.015*
BSA (m^2^)	0.34 (0.27–0.40)	0.30 (0.21–0.37)	0.022*
Height (cm)	64.50 (58.75–70.00)	61 (49.00–66.25)	0.030*
Weight (kg)	6.57 (4.52–8.42)	5.29 (3.43–7.16)	0.018*
Cross-clamp time (min)	83.19 ± 33.51	105.07 ± 38.64	0.013*
Cardiopulmonary bypass time (min)	111.33 ± 38.78	158.33 ± 57.60	<0.001**
ICU admission length (days)	2 (1–2)	6 (2.00–9.00)	<0.001**
Hospital admission length (days)	6 (5–10)	12 (9.00–17.00)	<0.001**
Pre-operative creatinine (umol/L)	24.52 ± 11.43	28.67 ± 10.33	0.185
Post-operative complications (*n*)			
Re-operation	2	6	
Wound infection	0	0	
Respiratory infection	2	9	
Atrial fibrillation	0	0	
Ventricular tachycardia	0	0	
Death	0	0	
Diagnosis (*n*)			
Aortic stenosis	0	1	
ASD	0	1	
AVSD	6	4	
Coarctation of the aorta	0	2	
DILV	2	1	
DORV	4	0	
HLHS	3	5	
Pulmonary atresia	1	2	
TGA	1	8	
ToF	1	6	
Tricuspid atresia	0	1	
Truncus arteriosus	1	3	
VSD	11	5	
Other	3	3	
Procedure (*n*)			
Arterial switch operation	1	7	
Atrial septal defect and ventricular septal defect repair	7	6	
Atrial septal defect surgery	0	1	
Atrial switch operation	0	1	
Coarctation repair surgery	0	2	
Glenn shunt	6	3	
Norwood operation	0	1	
Pulmonary valvuloplasty	1	0	
Double-outlet right ventricle repair	0	1	
Total anomalous pulmonary veins repair	1	3	
Repair of truncus arteriosus	1	4	
Subaortic stenosis resection	1	0	
Total repair of tetralogy of Fallot	1	6	
Ventricular septal defect repair	12	5	
Other	2	3	

Data displayed as mean ± standard deviation or median (interquartile range). AKI, acute kidney injury; BSA, body surface area (Mostellar formula); ICU, intensive care unit; ASD, atrial septal defect; AVSD, atrioventricular septal defect; DILV, double inlet left ventricle; DORV, double outlet right ventricle; HLHS, hypoplastic left heart syndrome; TGA, transposition of the Great Arteries; ToF, tetralogy of Fallot; VSD, ventricular septal defect. Two-sided *z*-tests and Mann–Whitney *U*-tests performed for normal and non-normal distributed data respectively. Significant *p*-values * <0.05, ** <0.01.

**Figure 1:**
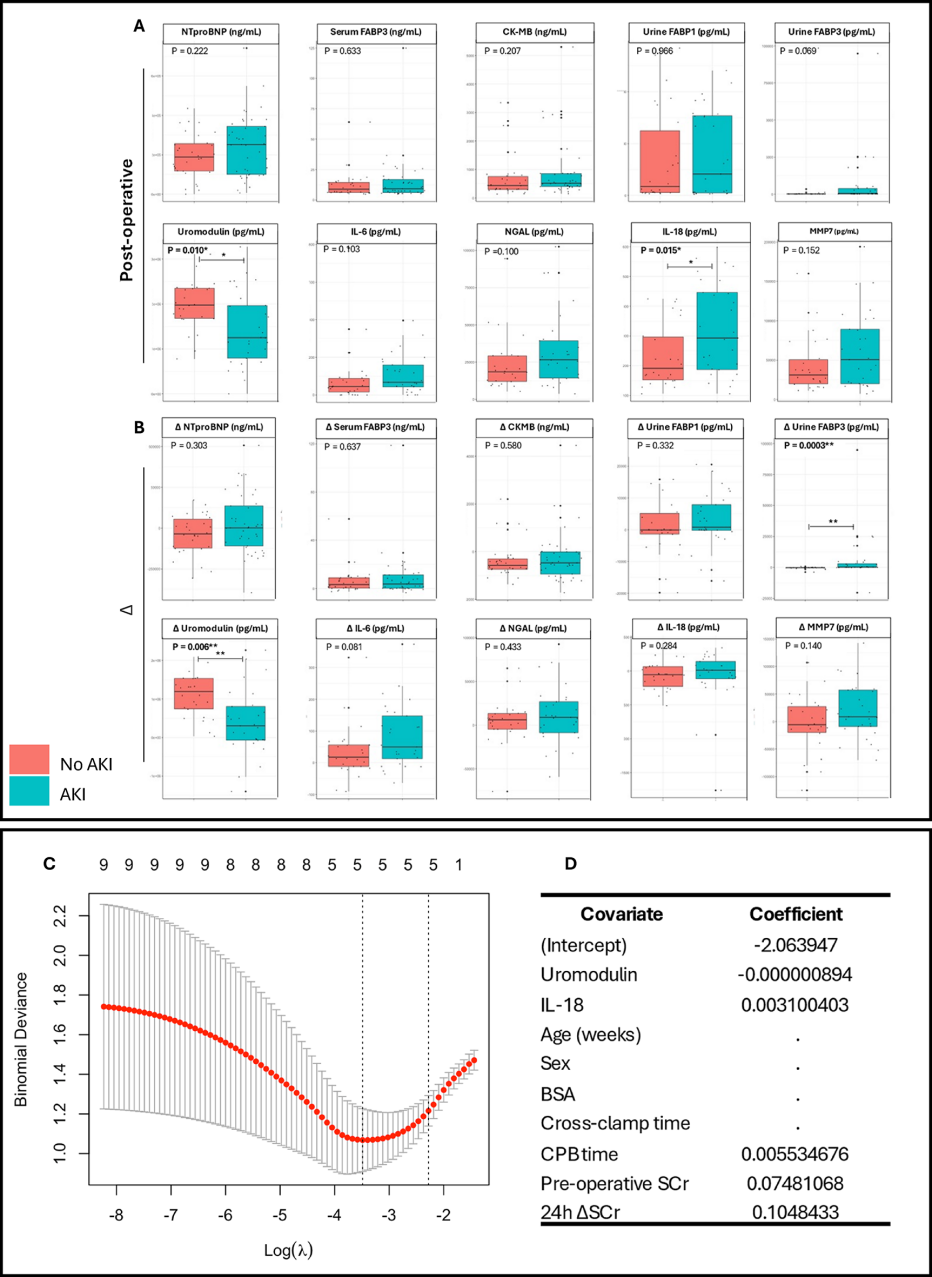
Comparison of urinary and serum clinical biomarker levels and the production of a composite LASSO regression model in pediatric congenital heart surgery patients stratified by postoperative acute kidney injury. Comparisons for each biomarker were performed on **(A)** biomarker levels at 24 h postoperatively, and **(B)** on the change in biomarker levels preoperatively to 24hrs postoperatively. **(C)** LASSO plot of binomial deviance against log lambda. **(D)** LASSO coefficient values for each covariate included within the compound model. BSA, body surface area; SCr, serum creatinine. Mann–Whitney *U*-tests performed for non-normally distributed data. Box-plots show median, inter-quartile range (IQR), and range with outlier values highlighted with dots. Significant *p*-values * <0.05, ** <0.01.

Biomarker levels were stratified by AKI status, postoperative urine IL-18 levels were higher in the AKI cohort (*p* = 0.014) conversely, uromodulin levels significantly lower (*p* = 0.010) (Figure 1A). Δ urine FABP3 was greater in the AKI cohort (*p* = 0.0003). Univariate analyses demonstrated urine uromodulin, urine IL-18, and serum FABP3 to be significantly associated with development of pCSA-AKI (*p* = 0.012, 0.040, and 0.042 respectively, Table 2). To address the issues of multicollinearity and overfitting, LASSO regression was conducted, further validating the potential biomarkers (Supplementary Figure S1B) and serum FABP3 (*p* = 0.321) was excluded.

**Table 2:** Single and multivariate logistic regression models for utility of prognostic biomarkers for the development of acute kidney injury in paediatric congenital heart surgery patients.

Biomarker	Estimate	Standard error	*Z*-value	*P*-value
Single biomarker models				
NTproBNP	1.86 × 10^−6^	3.06 × 10^−6^	0.608	0.543
CKMB	1.11 × 10^−4^	3.56 × 10^−4^	0.311	0.756
Serum FABP3	−4.29 × 10^−2^	2.11 × 10^−2^	−2.037	0.042*
Urine FABP1	2.68 × 10^−5^	4.17 × 10^−4^	0.643	0.52
Urine FABP3	−6.02 × 10^−5^	2.24 × 10^−5^	−0.268	0.788
Uromodulin	−1.94 × 10^−6^	7.60 × 10^−7^	−2.549	0.011*
IL-6	2.28 × 10^−3^	2.44 × 10^−3^	0.931	0.352
IL-18	7.39 × 10^−3^	3.59 × 10^−3^	2.056	0.040*
MMP7	1.69 × 10^−5^	1.03 × 10^−5^	1.635	0.102
NGAL	−5.91 × 10^−6^	1.94 × 10^−5^	−0.305	0.7606
Δ serum FABP3	−4.29 × 10^−2^	2.11 × 10^−2^	−2.033	0.042*
Δ uromodulin	−2.01 × 10^−6^	7.44 × 10^−7^	−2.704	0.007**
Δ IL-18	7.91 × 10^−5^	1.06 × 10^−2^	0.074	0.941
Compound model				
Intercept	−1.269	3.793	−0.335	0.7379
Urine uromodulin	1.87 × 10^−6^	9.52 × 10^−7^	−1.965	0.049*
IL-18	8.32 × 10^−3^	4.47 × 10^−3^	1.743	0.081
Serum FABP3	−4.47 × 10^−2^	4.51 × 10^−2^	−0.993	0.321
Intercept (Δ)	−7.166	5.46	−1.312	0.189
Δ uromodulin	−3.01 × 10^−6^	1.28 × 10^−6^	−2.357	0.018*
Δ IL-18	−2.9 × 10^−3^	2.13 × 10^−3^	−1.367	0.172
Δ serum FABP3	−2.94 × 10^−2^	4.13 × 10^−2^	−0.702	0.483

Estimate values, standard error, *z*-value, and *p*-value for each biomarker within the single biomarker models and compound models. Biomarkers that reached a threshold value of *p* < 0.1 were included in the compound model. Biomarkers that met the same threshold within the compound model were included in the LASSO compound model. Each model was adjusted for age, sex, BSA, cross-clamp time, CPB time, pre-operative SCR and 24 h Δ SCr. Significant *p*-values * <0.05, ** <0.01.

**Figure 2:**
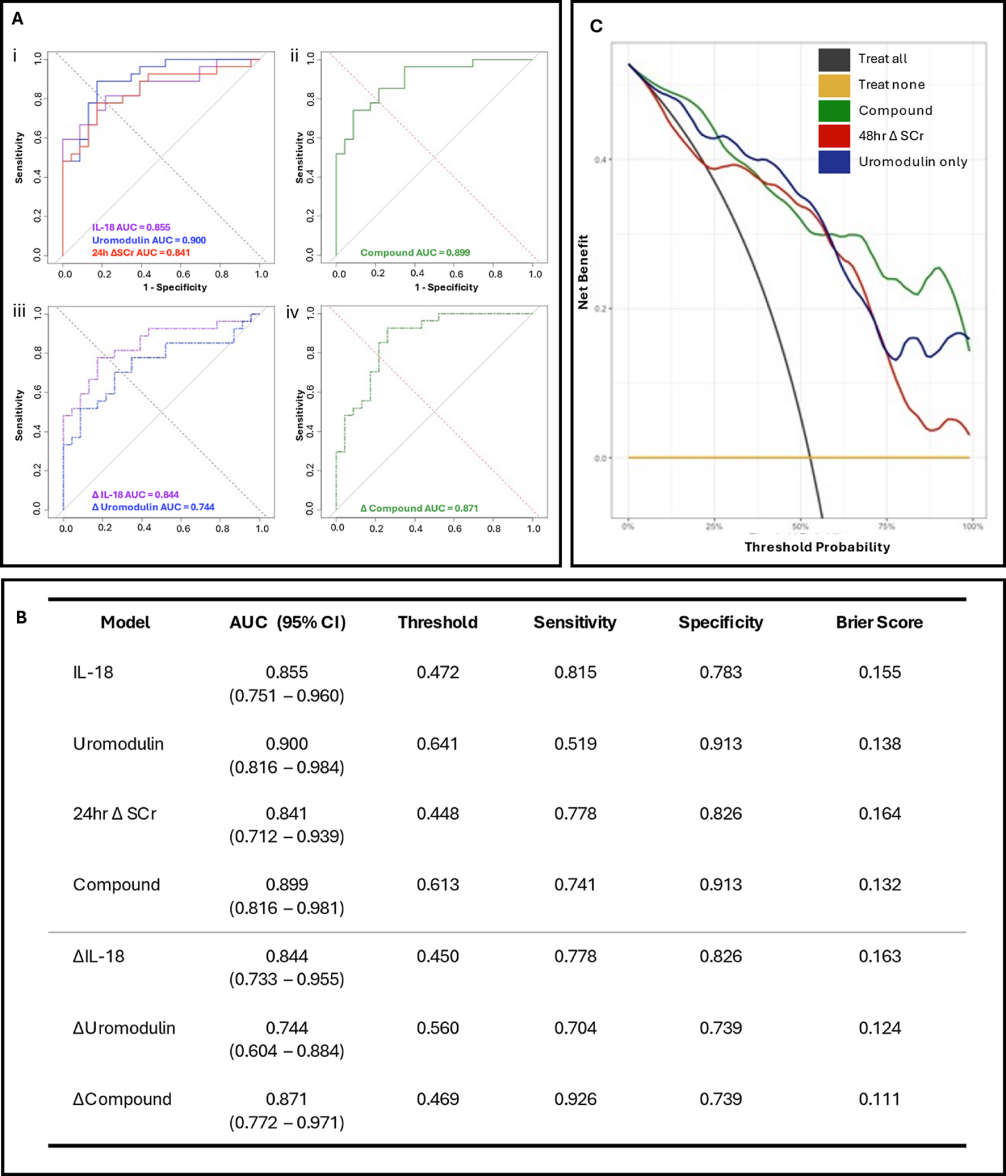
Prognostic models and decision curve analysis for postoperative acute kidney injury in paediatric congenital heart surgery patients. **(A)** Receiver operating characteristic (ROC) curves for; (i) postoperative IL-18 (purple), uromodulin (blue), and 24 h ΔSCr (red), (ii) composite postoperative biomarker (IL-18, uromodulin); (iii) 24 h hour postoperative change in IL-18 (Δ IL-18, purple dash) and uromodulin (Δ uromodulin, blue dash) and (iv) 24 h postoperative change composite biomarker (Δ IL-18, Δ uromodulin) model for LASSO regressions. **(B)** Evaluation of the bootstrapped single and multiple biomarker postoperative and delta models with ROC area under curve (AUC), threshold values, sensitivity, specificity, and Brier score. **(C)** Decision analysis curves comparing the novel compound model incorporating the urinary biomarkers IL-18 and uromodulin (green line), the uromodulin-only model (blue line) against a model using the change in creatinine from before the operation to 48 h postoperatively (48 h Δ SCr, red line). All models were adjusted for age (in weeks), sex, cross clamp time, CPB time, preoperative SCr level, and 24 h postoperative Δ SCr.

To confirm predicative capacity models were bootstrapped and evaluated using ROC analysis to determine AUC, threshold, sensitivity, and specificity (Figure 2A). The postoperative IL-18 model exhibited high precision with an AUC of 0.855 (CI 0.751–0.960). Comparatively, uromodulin exhibited an AUC of 0.900 (CI 0.816–0.984), with higher specificity (0.913 vs. 0.783) and lower sensitivity (0.519 vs. 0.815) than IL-18. Both postoperative single biomarker models had a greater AUC than the 24 h Δ SCr model (0.841). The compound model exhibited an AUC of 0.899 (CI 0.816–0.981), a threshold of 0.613, a sensitivity of 0.741, and a specificity of 0.913 (Figure 2B). To investigate the discriminability of IL-18 and uromodulin as early predictors of pCSA-AKI and associated ICU stay, the biomarkers were stratified by absolute value in an adjusted Poisson regression analysis (Supplementary Table S1). The highest postoperative IL-18 concentrations had a significant effect on ICU stay duration in comparison to the lowest (Model 1; OR 1.88, *p* < 0.0001, Model 2; OR 2.18, *p* < 0.0001). Furthermore, median ICU stay durations significantly differed between postoperative IL-18 tertiles (*p* = 0.02).

Decision curve analysis was conducted on both the postoperative compound and uromodulin models in comparison to a fully-adjusted model incorporating the 48 h postoperative Δ SCr (Figure 2C). Integration of the biomarkers demonstrated comparable net clinical benefit to the 48 h Δ SCr model at lower threshold probabilities. Both the compound and Uromodulin models demonstrated comparable net clinical benefit to the 48 h Δ SCr model at lower threshold probabilities (i.e., patients at a higher risk of pCSA-AKI with whom clinicians may prefer a “treat all” approach).

## Discussion

Infants below the age of 12 months have the highest rates of pCSA-AKI (4, 7), partially due to undeveloped parenchyma, making these patients more vulnerable to ischaemic injury (23), making them a unique high-risk group. Recent studies have demonstrated the utility of serum biomarkers to improve diagnosis of AKI in adults (24–26) however obtaining blood samples from the smallest patients limits this application. Uromodulin and IL-18 perform comparatively well when measured at a single timepoint (24 h post-operatively) compared to measuring the change in biomarker concentrations following surgery. This greatly improves the clinical utility and practicality of the model, requiring the collection of fewer samples, reducing collection and processing time.

When directly compared to a model utilising the Δ SCr at 48 h post-operatively (representing current diagnostic approaches), a decision curve analysis demonstrated superior clinical benefit particularly at higher threshold probabilities where diagnostic uncertainty may be present. At higher threshold probabilities the compound model demonstrated a greater net clinical benefit than the Δ SCr model, which may suggest that the greatest utility of this biomarker model may be in patients where there are high levels of diagnostic uncertainty and ambiguity. Moreover, since the two most robust biomarkers were both obtained from urine samples, the models avoid the need for repeat, difficult venepuncture. The recent development of novel point-of-care urine biomarker assays such as RenaStick (24), which measures urine concentrations of kidney injury molecule 1 (KIM-1), and NephroCheck (Astute Medical, Inc.) (25), which measures 2 urine biomarkers; tissue inhibitor of metalloproteinase 2 (TIMP-2) and insulin-like growth factor binding protein 7 (IGFBP-7), highlights the potential for the development of a similar rapid, point-of-care urine test incorporating Uromodulin and IL-18.

Here we suggest the utilization of rapid urine tests to identify early pCSA-AKI within 24 h of surgery, facilitating prompt intervention with the potential to mitigate the most serious sequelae of renal injury. Whilst this pilot study was limited to a small cohort, our finds represent a first step into the identification of robust AKI biomarker discovery, with larger, multi-centre studies able to address the limiting factors in future work. Although internal validation of the models was performed through bootstrapping, external validation of the models would ideally be performed in a separate, multi-ethnic, multi-national patient population. Additionally, whilst the AUC for the models was sizeable, the sensitivities in the ROC analyses were modest. One limitation of DCA is that these analyses do not take into consideration the differential importance of false negative and false positive decisions. Furthermore, this study measured biomarker concentrations at two timepoints; 24 h pre- and post- operatively. Moreover, as this study defines AKI as a serum creatinine increase 48 h post-surgery, due to the ability to obtain samples whilst in intensive care with lines available, patients who develop AKI between day 2–7, in line with the KDIGO guidelines (7), are missed from the cohort. Future longitudinal studies should measure Uromodulin and IL-18 levels at multiple timepoints post-operatively to establish an optimum diagnostic window, as well as investigate whether these biomarkers can monitor disease progression. Despite these limitations, this study provides further insight into the potential roles of urine biomarkers such as Uromodulin and IL-18 in the diagnosis of pCSA-AKI.

## Data Availability

The raw data supporting the conclusions of this article will be made available by the authors, without undue reservation.
